# Correction: Legal Frameworks Upholding Deceased Individuals’ Rights and Enabling the Use of Cadavers in Anatomy Education and Research: A Systematic Review

**DOI:** 10.7759/cureus.c451

**Published:** 2026-06-15

**Authors:** Sundip Charmode, Lalit Ratanpara, Nishat Sheikh, Kumar Satish Ravi, Simmi Mehra

**Affiliations:** 1 Anatomy, All India Institute of Medical Sciences Rajkot, Rajkot, IND; 2 Forensic Medicine and Toxicology, All India Institute of Medical Sciences Deoghar, Deoghar, IND; 3 Anatomy, All India Institute of Medical Sciences Gorakhpur, Gorakhpur, IND

This article has been corrected to specify that the Malawi Anatomy Act, not the Africa Anatomy Act, was updated and revised in 2016. No continent-wide African anatomy statute currently exists.

